# Neurofilament Light Chain and Multiple Sclerosis: Building a Neurofoundational Model of Biomarkers and Diagnosis

**DOI:** 10.3390/neurolint17040056

**Published:** 2025-04-11

**Authors:** Rany Aburashed, Ansam Eghzawi, Daniel Long, Robert Pace, Ali Madha, Jeanie Cote

**Affiliations:** 1 Insight Hospital and Medical Center, Chicago, IL 60616, USA; 2 Neurogen Biomarking LLC, Dover, DE 19901, USA; 3 Memorial Healthcare, Owosso, MI 48867, USA; 4 Insight Institute of Neurology and Neurosurgery, Flint, MI 48507, USA

**Keywords:** multiple sclerosis, biomarker, neuroinflammation, relapsing MS, neurofilament light chain (NfL)

## Abstract

Neurofilament light chain (NfL), an abundant cytoskeletal protein in neurons, has emerged as a promising serum biomarker that indicates non-specific neuronal damage secondary to various neurologic diseases, including multiple sclerosis (MS). Emerging evidence suggests that serum NfL levels correlate with future disability, brain atrophy, predict new disease activity, and decrease in response to various disease-modifying therapies. As research continues to validate NfL’s potential role in clinical practice, the need for a practical model to conceptualize and visualize its relevance to MS pathology becomes evident—not only for healthcare providers but also for patients. To address this, we propose the Neurofoundational Model (NFM), which likens a neuron to a home, with various parts of the home representing distinct regions of the central nervous system (CNS). In this model, the home (neuron) experiences scenarios such as a fire, an earthquake, and a slow flood, representing distinct MS disease states. A fire illustrates an MS relapse with good recovery, where serum NfL levels rise during the relapse and subsequently return near baseline. An earthquake represents an MS relapse with poor recovery, where NfL levels increase and remain elevated above baseline. Finally, a slow flood depicts MS in progressive stages, characterized by sustained and gradually increasing serum NfL levels without abrupt clinical changes. This approach offers a clear and relatable visualization for clinicians and patients alike, illustrating the dynamics of serum NfL levels during CNS damage caused by demyelination. By integrating this model into clinical practice, we aim to enhance understanding and communication regarding the role of NfL in MS pathology and its potential utility as a biomarker.

## 1. Introduction

Multiple sclerosis (MS) is a chronic autoimmune inflammatory disease affecting the central nervous system (CNS) [1]. Both the chronicity and heterogeneity of the disease present clinicians with the challenge of trying to monitor disease tempo, predict disability, and evaluate response to disease-modifying therapies (DMT) [2]. To date, MRI presents neurologists with the strongest monitoring tool; however, even with advanced sequences, this modality still has its shortcomings [3]. Saying this, a reliable, objective, and easily obtainable biomarker that can augment MRI findings remains highly desirable. Research in recent years has focused on improving knowledge about which biomarkers are reliable to improve the management of MS patients [4]. These biomarkers can be categorized into different types, including imaging biomarkers, molecular biomarkers, and neurophysiological biomarkers [5]. Neurofilament light chain (NfL) is a structural protein that is predominantly found in the neurons and has emerged as a potential biomarker for MS [6]. Studies showed that elevated NfL in the serum and CSF was found to correlate with the disease severity making it an important tool to be used in the clinical practice [7,8]. Quantification of NfL levels is promising in diagnosing and monitoring MS, as well as various neurologic conditions including neurodegenerative disorders, CNS vascular insults, and motor neuron disease [9,10,11,12,13,14]. Several studies have supported the paradigm that serum NfL levels may further assist in the management of MS by helping predict new disease activity, correlating with future brain atrophy, portending poorer prognosis, and by assessing response to DMT [15,16,17,18,19,20,21,22].

As the future possibility of integrating NfL into clinical practice is becoming more realistic, a theoretical model is desirable to help conceptualize the connection between NfL serum levels (indicating axonal damage) and the clinical pathology seen in patients. A simplified conceptual tool for MS educators and clinicians is necessary to facilitate discussions on NfL role and dynamics. As such, we propose the Neurofoundational Model detailed in this article.

## 2. Methods

This perspective study presents a conceptual model for understanding the role of neurofilament light chain (NfL) in multiple sclerosis. A targeted literature review was conducted using electronic databases, including PubMed, Scopus, and Web of Science, up to December 2024. Keywords used in the search included “multiple sclerosis”, “biomarkers”, “neuroimaging”, “molecular markers”, “immunological markers”, “diagnosis”, and “neurofilament light chain”. Articles were selected based on their relevance, quality, and recency. The selection included original research articles, reviews, and meta-analyses that specifically focused on the neurofilament light chain (NfL) role in multiple sclerosis. Data were extracted regarding the types of biomarkers, their clinical applications, and the findings from various studies.

## 3. Discussion

### 3.1. Neurofilament Light Chain: Structure and Function

**Figure 1:**
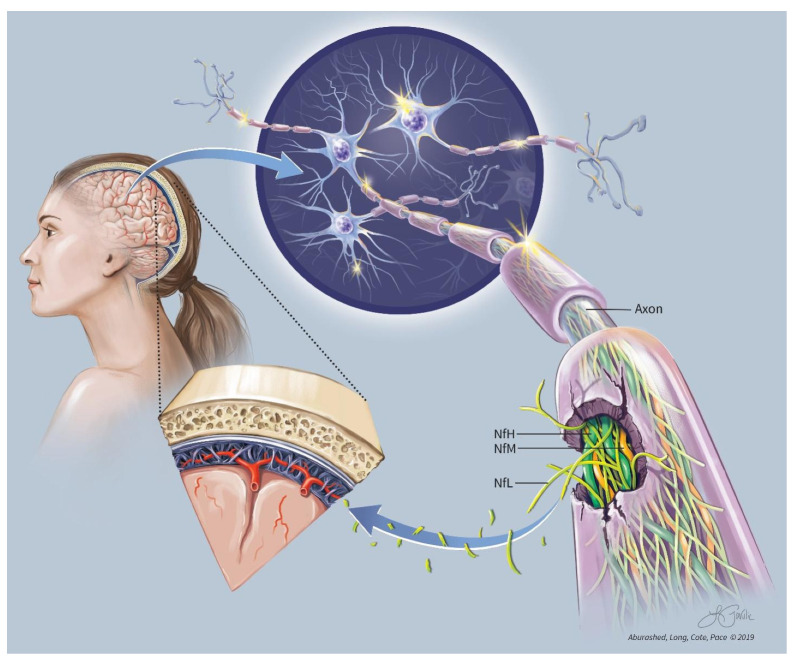
NfL Conceptual.

Neurofilaments (Nf), composed of various combinations of light chain (NfL), medium chain (NfM), and heavy chain (NfH) subunits, are the most abundant structural proteins that make up the cytoskeletal support in large, myelinated neurons [23]. Running parallel to the axon itself, Nf are directly responsible for the radial growth of the axon, increasing the diameter up to 10-fold, and thus, indirectly playing a role in conduction velocity [24,25]. The three major neurofilament subtypes (light, medium, and heavy), so-named based on molecular mass, combine to form heteropolymers with NfL serving as the core in addition to being the most abundant component. (Figure 1) [26,27]. Given this relative abundance in relation to other neurofilaments, NfL is an appealing choice as a biomarker for axonal damage. For simplification, if axons are thought of as a home, the neurofilament-rich cytoskeletal matrix could be thought of as the foundation on which the home was built.

### 3.2. Potential Clinical Applications of Assessing NfL in MS

NfL is a protein of the neurofilament network which is a part of the neuronal cytoskeleton. It is found mainly in the large, myelinated axons and is released into CSF and blood during neuroaxonal injury which is central in the MS pathogenesis [28,29]. NfL is found to be a promising tool for MS activity monitoring because it is considered a highly sensitive and specific marker for neurodegeneration [30]. Elevated CSF and blood levels of NfL have been associated with MS activity, relapses, disability progression, and lesions on MRI [31]. NfL release to CSF is linked to neuronal or axonal damage which is characteristic of MS [32]. It provides a direct picture of neuroaxonal injury which makes it a unique tool for assessing the neurodegenerative part of MS [33].

When axonal damage occurs, regardless of cause, these structural proteins are leaked at various concentrations within the CNS. As these filaments are leaked, they make their way to the CSF and serum. CSF concentrations occur on the order of hundreds to thousands of picograms per milliliter, while serum concentrations are roughly 10-fold less [34,35]. Early measurements of NfL levels, obtained via standard enzyme linked immunosorbent assays (ELISAs), were limited to CSF samples given that the relatively low concentrations of NfL were at the end of the detection capability for most traditional assays. After the advent of future generation ELISAs, reliable serum NfL levels could be obtained [36,37]. Advancements in the technology of assays to measure NfL improved its accessibility and sensitivity. The use of ultra-sensitive immunoassays such as single molecule array (SIMOA™) technology gave accurate measurements of NfL in both serum and CSF samples making it a practical tool for routine clinical practice [8,35]. SIMOA™, as the name implies, is able to quantify and reliably report data to the level of single molecules in the serum, thus providing up to one-thousand-fold higher sensitivity than traditional ELISAs [38,39]. Once this type of technology became available, various datasets have shown that there is a high correlation between CSF and serum NfL levels [40].

Recent studies have suggested that blood-based NfL measurements, although less invasive than CSF analysis, can still provide reliable information regarding disease activity and prognosis in MS [6].

An MRI of the brain remains informative in showing the damage occurring to the structure of the “home”, whereas NfL testing may serve as an indicator of the true degree of damage occurring on a “foundational level”, below what is detected radiographically. Several studies have shown that NfL levels among other biomarkers are suggestive of more disease progression, especially in the acute phase [40]. An observational study on 257 MS patients found that NfL levels were a significant marker for disease progression and can be utilized as a tool to stratify patients according to disease progression, for both clinical trials and clinical practice [41]. Other clinical studies in the current literature also supports NfL as a biomarker being able to offer objective information to help predict new disease activity, signal a potential increased risk for future disability, and monitor response to treatments [16,18,19,20,21].

Multiple studies have reported that the elevated NfL is predictor for poor clinical outcomes in MS [42]. Kuhle et al. (2016) showed that NfL levels measured in blood and CSF are significantly higher in patients with relapsing forms of MS compared to healthy controls and patients with primary progressive MS (PPMS), and these levels tend to decrease with effective disease-modifying therapy (DMT) [43]. In RRMS, NfL levels were useful in disease activity monitoring during and after the relapse indicating an ongoing axonal damage [44]. Because NfL levels were also high in CIS, this indicates its utility to predict the conversion to clinically definitive MS [45].

Furthermore, NfL has been shown to be a more reliable biomarker for predicting disease progression in progressive forms of MS compared to conventional MRI parameters, such as T2 lesion load or the presence of gadolinium-enhancing lesions [3]. This reflects the fact that NfL correlates with axonal damage rather than just inflammatory activity, which may be less pronounced in progressive MS stages [29]. Studies reported NfL as a prognostic marker for MS due to its ability to reflect both clinical and subclinical disease activity and its correlation with changes in disability scores with more advanced levels were linked with greater long-term disability [46]. In addition, the therapeutic efficacy and response to disease-modifying therapies were tracked using longitudinal monitoring of NfL especially in progressive types of MS where clinical improvement tends to be less apparent [47]. For example, in patients treated with high-efficacy DMTs like natalizumab or ocrelizumab, NfL levels tend to decrease, indicating a reduction in neuroaxonal damage, even when clinical symptoms remain stable [48]. However, in patients with more progressive forms of MS or those on lower-efficacy therapies, NfL levels may remain elevated, signaling ongoing axonal damage and poor prognosis [49].

Despite these advances, challenges remain in standardizing NfL measurement across different labs and understanding the full range of physiological and pathological conditions that may influence NfL levels [50].

Even though elevated NfL levels are suggestive of disease activity and progression, they are still non-specific numerous neurologic diseases damage axons and thus, result in elevated levels, as NfL has potential utility in other neurodegenerative diseases like Alzheimer’s disease, Parkinson’s disease, and amyotrophic lateral sclerosis [14]. Therefore, its role in distinguishing between different types of neurodegenerative diseases remains an area of ongoing research [51].

Moreover, some non-neurological reasons may result in an increase in NfL levels such as elevated blood pressure, diabetes, and aging [52]. Thus, NfL levels alone are not enough to be used as a diagnostic tool and the utility in NfL as a biomarker for MS is suggested after a confirmed diagnosis and clinical evaluation to rule out contributions from age and other potentially confounding comorbidities [53]. Following a confirmation of the diagnosis and the standard of care clinical evaluation, NfL levels can be utilized for disease monitoring and prediction of progress. As such, we propose the Neurofoundational Model to serve as a teaching aid to clinicians and patients alike.

### 3.3. A Model of Understanding NfL in MS

What has emerged from the literature thus far is a correlation of serum NfL levels corresponding to axonal damage and the potential value that this correlation has in terms of making NfL a biomarker for neurologic disease monitoring. What has yet to be described in the literature is a model to conceptualize the process of how to connect axonal damage and NfL levels to the clinical picture; as a result, we propose the Neurofoundational Model, or NFM for NfL. It is important to emphasize that this model is a theoretical construct. Every vigorous attempt was made to support this model with our current understanding of NfL as obtained by the existing studies. However, at times this model does make certain assumptions that will be discussed when appropriate. At present, we have no evidence to refute our assumptions, but confirming their validity may serve as a basis for future studies.

While the Neurofoundational Model was designed to conceptualize NfL’s role specifically in MS, we acknowledge that elevated NfL levels are also observed in other progressive neurological disorders, such as Alzheimer’s disease, ALS, and Parkinson’s disease. As mentioned earlier, these conditions involve ongoing neuroaxonal injury, which could theoretically align with the “flooding” concept in our model. However, unlike MS, these diseases lack a relapsing-remitting pattern, and their distinct pathophysiology limits direct comparisons. Further exploration is needed to determine whether a modified version of this model could effectively illustrate NfL dynamics in these conditions. For this perspective, we focus on MS, where NfL’s clinical significance continues to grow.

The Neurofoundational Model is designed as a theoretical illustration to help with conceptual understanding rather than provide a direct validation of NfL’s clinical applications. One of the key limitations is that the model has not been tested through quantitative clinical studies, and its applicability in clinical patient care is yet to be determined. Further research is necessary to assess the correlation between NfL levels and the disease scenarios shown in the model.

Additionally, while the model serves as a tool for patient and clinician education, it simplifies the complexity of MS pathophysiology. It also does not account for all variables influencing NfL levels, such as age, comorbidities, and treatment effects.

It is important to highlight that although our model focuses on MS as the disease process leading to axonal damage, our model is not intended to outline MS phenotypes or help conceptualize the disease course as per the already existing topographical model proposed by Krieger et al. [54].

**Figure 2:**
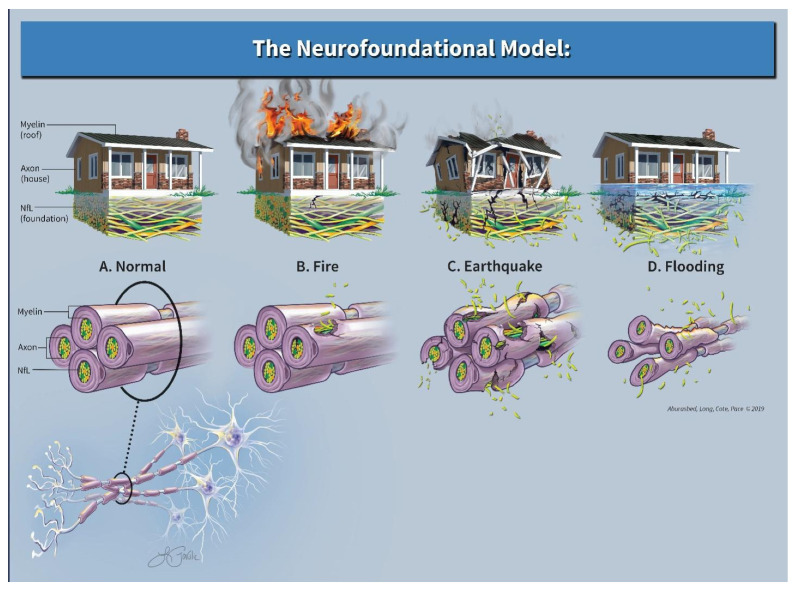
Neurofoundational composite.

For the Neurofoundational Model, we use the analogy of a home in three different scenarios (see Figure 2). Within the CNS, consider the myelin as a roof, the axons as a house, and the cytoskeletal matrix, namely NfL, as the foundation on which that home is maintained. As the roof (myelin) and the home (axons) are damaged, our current imaging techniques are quite adept at revealing predominately evidence of damage to the roof and to a lesser extent the house. What standard imaging technique s miss, however, is damage to the neuron’s foundation. This damage is perhaps an equally or more important phenomenon to assess as evidenced by the occurrence of a clinical-radiographic disconnect, i.e., a patient that clinically appears “normal”, but has an MRI that would suggest otherwise or vice versa.

#### 3.3.1. Scenario 1

Imagine a home that was on fire with flames consuming the roof and additional evidence of burn marks clearly visible to the outer façade. Aside from this superficial inspection, the foundation remains largely intact and outside of the obvious cosmetic repairs, the home may return to its normal state with time. Neurologically speaking, this may be akin to a patient with MS who suffers an acute exacerbation, has an MRI revealing several new enhancing lesions, yet eventually has excellent recovery. In terms of NfL levels, the current literature supports the idea that the corresponding serum NfL level would be higher than past baseline values; however, this elevation would be transient and may return to near pre-relapse levels after time, thus signifying that “as the dust settles”, there is a potential for full recovery [16,34,50,55]. What our model assumes is that this initial acute increase in the patient’s NfL level would be relatively lower than NfL levels during a relapse with poor recovery. Clinically, the patient has neurologic dysfunction, yet complete recovery occurs over time. Radiographically, the areas of neuronal damage were evident, but the extent of the damage was not. In this theoretical case, given a relatively small and transient increase in NfL levels from the baseline, the foundation remained relatively intact and was able to support eventual neuronal repair.

#### 3.3.2. Scenario 2

Imagine a second home hit by a large earthquake. In this scenario, there would be damage to the entire property: roof, home, and foundation. In our model, this would be represented by both abnormal imaging as well as a substantial and sustained elevation of serum NfL. An example of this would be the previously mentioned patient with MS who has similar MRI findings and similar clinical symptoms, however with a much higher elevated NfL level (as opposed to the first scenario) that remains elevated when compared to prior baseline. Again, the current literature supports the notion that as the “aftershocks” stop, a new higher baseline NfL level may portend a poorer prognosis [17,20,22]. Given that the patient’s cytoskeletal foundation is more severely damaged, poor recovery ensues and permanent disability may occur.

#### 3.3.3. Scenario 3

Lastly, imagine a third home that is slowly being flooded from below. Cosmetically the home looks relatively unadulterated; however, structurally the foundation slowly erodes. This scenario is similar to a patient with progressive forms of MS. Clinically, this theoretical patient would deny any abrupt changes, but rather endorse a slow deterioration in function (i.e., slowed gait, emergence of bladder dysfunction, impaired dexterity, progressive worsening of old symptoms). Imaging of the brain may be relatively normal (especially early on in disease course) or essentially stable in terms of lesion count; however, elevations in NfL may serve as an indicator of an otherwise unobserved foundational loss that is insidious and progressive. Studies seem to support this concept as data exists and correlates future atrophy and future disability with elevated serum NfL levels early in the disease course [20].

The Neurofoundational Model reflects the heterogeneity of MS progression. While not all patients who experience severe relapses transition to SPMS, the presence of persistently elevated NfL levels may serve as an early indicator of future progression. This distinction highlights the model’s potential clinical utility in interpreting NfL trends over time.

Studies have shown that elevated serum NfL levels correlate with disease activity, progression, and neuroaxonal damage, supporting the basis of each scenario in our model.

In Table 1, we summarize key findings that correspond to the different MS stages represented by the model.

## 4. Conclusions

The Neurofoundational Model was intentionally designed as a simplified conceptual tool for MS educators and clinicians to facilitate discussions on NfL dynamics rather than as a comprehensive pathophysiological model. It uses MS as an example of a CNS disease that damages neurons to help highlight our primary focus, conceptualizing what is happening to NfL levels during this damage.

In terms of assuming that NfL levels in acute relapses correlate with recovery, the data will likely be forthcoming. However, even if this assumption does not hold true, current evidence does support that a new post-relapse NfL baseline obtained weeks after the event does aid in prognostication. Aside from this, we recognize that our Neurofoundational Model using the examples of a home to describe the connection of NfL to neuronal damage represents a simplification of the intricate and complex biological processes. We realize that in many cases of MS, patients have evidence of both occult “flooding”, as well as superimposed “fires” and/or “earthquakes” indicating ongoing insidious progression with superimposed inflammation. It was not our goal to describe the microscopic intricacies of the processes that are occurring on a molecular level, but rather to help provide a macroscopic and concrete visualization of how to clinically view the potential utility of NfL. Extensive data are still being collected to further understand the implications of NfL as a biomarker in clinical practice. Aside from the above, our secondary goal was to foster an appreciation towards the exciting potential of NfL measurements in the future. It remains imperative to note that our current approach to NfL as well as the SIMOA assay remains in the investigational state. Regardless, the future potential of using this biomarker in the clinical setting is exciting.

## Figures and Tables

**Table 1:** Comparison of MS Disease Stages and Corresponding Neurofoundational Model Scenarios with the Supporting Literature.

Scenario	MS Stage Represented	Supporting Evidence
Scenario 1: Fire (Acute Inflammation with Recovery)	Relapsing-Remitting MS (RRMS) with Good Recovery	Studies have shown that NfL levels spike during relapses but return to near-baseline levels in cases of full recovery. This transient elevation reflects acute axonal injury that resolves with effective remyelination or repair (Varhaug et al., 2018 [16]; Disanto et al., 2017 [34]).
Scenario 2: Earthquake (Severe Relapse with Residual Deficits)	RRMS with Poor Recovery/Possible Transition to SPMS	Persistent elevation of NfL following a relapse is associated with worse outcomes, increased disability progression, and brain atrophy, suggesting that this scenario represents patients with aggressive RRMS or those transitioning toward SPMS (Kuhle et al., 2019 [6]; Chitnis et al., 2018 [17]).
Scenario 3: Flooding (Progressive Degeneration)	Secondary Progressive MS (SPMS) and Primary Progressive MS (PPMS)	Chronic and sustained elevations in NfL correlate with progressive neurodegeneration, even in the absence of acute relapses. This aligns with the “flooding” concept, representing axonal loss that continues independently of overt inflammatory activity (Leppert et al., 2022 [3]; Kapoor et al., 2020 [49]).
